# DNA hypomethylation of the COX-2 gene promoter is associated with up-regulation of its mRNA expression in eutopic endometrium of endometriosis

**DOI:** 10.1186/2047-783X-17-12

**Published:** 2012-05-18

**Authors:** DanBo Wang, Qi Chen, ChiYuan Zhang, Fang Ren, Tong Li

**Affiliations:** 1 Department of Obstetrics & Gynecology, Shengjing Hospital Affiliated to China Medical University, Shenyang, 110004, People’s Republic of China; 2 Department of Obstetrics & Gynecology, Shengjing Hospital Affiliated to China Medical University, 36 Sanhao Street, Shenyang, 110004, People’s Republic of China

**Keywords:** Endometriosis, DNA hypomethylation, COX-2 mRNA expression, Epigenetics

## Abstract

**Background:**

Accumulated evidence reveals that cyclooxygenase-2 (COX-2) was overexpressed in eutopic endometrium of endometriosis, which may play a critical role in the pathogenesis of endometriosis. However, few studies have been performed to explore the molecular mechanisms underlying the abnormal high expression of COX-2 in endometriosis. Considering the fact that a number of recent studies have shown DNA methylation affecting some genes in endometriosis, the present study was therefore aimed to determine whether the observed high expression COX-2 in endometriosis is caused by the hypomethylation of CpG island within the promoter of this gene.

**Methods:**

The endometrial tissues were collected from 60 women with endometriosis (endometriosis group) and 20 women without endometriosis (control group). The methylation status of COX-2 was examined by methylation specific PCR. Quantitative real-time RT-PCR was performed to measure COX-2 mRNA level in endometrial tissues.

**Results:**

The frequency of promoter hypermethylation of COX-2 was lower in eutopic endometrium of the endometriosis group (41.7%) than that in the control group (75.0%), *P* < 0.05. COX-2 mRNA level in the eutopic endometrium of the endometriosis group was 2.61-fold higher than that in the control group (*P* < 0.01). COX-2 mRNA level in unmethylated endometrium of the endometriosis group or the control group was 2.39-fold and 2.66-fold, respectively, higher than that in the methylated endometrium of the same group (*P* < 0.01).

**Conclusions:**

The hypomethylation within the promoter of COX-2 may be responsible for the elevated gene expression in eutopic endometrium of endometriosis.

## Background

Endometriosis is an estrogen-dependent gynecological disorder that affects 6-10% of women of reproductive age. It is characterized histologically by the presence of endometrial tissue at sites outside of the uterine cavity, primarily on the pelvic peritoneum and ovaries, resulting in severe pelvic pain, pain during intercourse, and infertility [1,2]. To date, the etiology and pathogenesis of endometriosis remain largely unknown. Endometriosis is a benign gynecological disease with malignant behaviors, such as enhanced proliferation and cell invasion, ectopic implantation of distant organs similar to the tumor metastasis. The eutopic endometrium of patients with endometriosis has various alterations compared with endometrium of healthy women [3]. Aberrant expression of genes in eutopic endometrium was reported be involved in cell adhesion, invasion, and angiogenesis, therefore it was quite critical to the pathogenesis of endometriosis [4-6].

The ectopic endometrium of endometriosis often behaves unpredictably; it can vary from microscopic foci to large, grossly visible, endometriotic cysts, which leads to difficulties in research, diagnosis, and treatment. Eutopic endometrium of endometriosis is readily available and gene alteration in the eutopic endometrium can be easily detected. Identification endometriosis-related genes in eutopic endometrium will further reveal the pathogenesis of endometriosis and offer the basis for targeted gene diagnosis and therapy of endometriosis. In the previous study, we identified 10 up-regulated genes in the eutopic endometrium of endometriosis during the secretory phase using cDNA-RDA and found that cyclooxygenase-2 (COX-2) was one of the up-regulated genes [7]. As the key enzyme in the conversion of arachidonic acid to prostaglandins (PGs), COX-2 can be induced by growth factors, oncogenes, and tumor promoters, and has been mainly associated with the inflammatory response [8]. Its elevated expression in the eutopic endometrium has also been reported to be associated with endometriosis [9,10]. However, the underlying mechanism of overexpression of COX-2 in eutopic endometrium of endometriosis has not been well defined.

DNA methylation is an epigenetic phenomenon known to play a critical role in the regulation of gene expression in development, differentiation, and complex diseases, with cancer being the most prominent example [11,12]. Moreover, aberrant methylation of promoter CpG island of the COX-2 has been known as an alternative mechanism of its abnormal expression and contributes to the carcinogenesis in many human cancers [13,14]. Recently, DNA methylation has also been shown to affect a number of genes in endometriosis [15]. These findings lead us to investigate whether aberrant expression of the COX-2 in eutopic endometrium of endometriosis is caused by aberrant methylation of the COX-2 CpG island. The nuclear factor responsible for the interleukin-6 expression (NF-IL6) site as one of the critical cites of the COX-2 promoter plays an important role in the regulation of COX-2 expression [16]. In the present study, we investigated, for the first time, that whether the observed elevated expression of the COX-2 gene in endometriosis is associated with the hypomethylation of NF-IL6 site within the promoter of this gene.

## Methods

### Patients and specimens

Eutopic endometrium samples were collected from 60 patients with an average age of 43.65 ± 3.99 years, who underwent hysterectomy due to endometriosis stages III and IV according to the Revised American Fertility Society Classification for Endometriosis at the Department of Obstetrics and Gynecology, Shengjing Hospital of China Medical University. For controls, endometrium samples were obtained from 20 women with an average age of 43.20 ± 2.87 years, who underwent total hysterectomy due to cervical intraepithelial neoplasia III in the same hospital, surgically confirming without endometriosis. Diagnosis was confirmed with histopathological examination in all cases. All subjects presented regular menstrual cycles (cycle length was approximately 25 to 32 days). Cycle stage was estimated according to the date of the last menstrual phase or by histological evaluation [17]. All specimens were obtained in the secretory phase of the menstrual cycle (days 15 to 28) in our study. As shown in Table 1, there was no difference between the two groups with respect to the cycle phase. None of these patients had received any GnRH analogue, antibiotics, radio-, chemo-, or hormone therapy in the last 6 months prior to the surgery. Endometrium samples were gathered within 10–15 min after hysterectomy and immediately frozen in liquid nitrogen and then preserved in −80°C refrigerator until further use. Written informed consent was obtained before surgical procedures, including a consent form and protocol approved by the Institutional Review Boards of China Medical University. 

**Table 1:** The cases of the endometriosis group and the control group in different phase of menstrual cycle (n)

**Phase of menstrual cycle**	**EMs group**	**Control group**	***χ*2**	***P***
Early secretory	20	7	0.032	0.984
Mid secretory	27	9		
Late secretory	13	4		

### Methylation specific PCR (MSP)

Genomic DNA was extracted from endometrial tissues by using the TIANamp Genomic DNA Kit (Tiangen Biotech Co., Ltd., Beijing, China) according to the manufacturer’s instructions. Then, 1 μg genomic DNA was modified by sodium bisulfite with the CpGenome^TM^ DNA Modification Kit (Chemicon, Billerica, MA, USA). This modification converts unmethylated cytosine to uracil and leaves 5-methyl cytosine unchanged. Briefly, 20 μL of genomic DNA was treated with 550 μL of a mixed solution of 3.5 M sodium bisulfite/1 mM hydroquinone (pH 5.0). After incubation at 55°C for 16 h, the treated DNA was purified and desulfonated with 0.3 M NaOH. The modified DNA obtained was ethanol-precipitated and dissolved in 50 μL of Tris-EDTA buffer. The primers used for the methylated COX-2 gene-promoter regions were as follows: (a) COX-2 F, 5^′^-GAAGCGTTCGGGTAAAGATTGC-3^′^ and (b) COX-2R, 5^′^-AAATTACGTAAACCCGATAAAA-3^′^. Primers for unmethylated COX-2 were as follows: (a) COX-2 F, 5^′^-TGGAAGTGTTTGGGTAAAG-3^′^ and (b) COX-2R, 5^′^-AAAATTACATAAACCCAATA-3^′^. To exclude false-positive and false-negative results, universal unmethylated DNA and universal methylated DNA were purchased from Chemicon and served as controls.

### Quantitative real-time RT-PCR

Total RNA was isolated by using the RNAsimple total RNA kit (Tiangen, China) according to the manufacturer’s instructions. One microgram total RNA was reverse transcribed using the TIANScript RT Kit (Tiangen, China). Quantitative real-time RT-PCR was performed using SYBR green (Tiangen, China) on Exicycler^TM^™ 96 Real-Time Quantitative Thermal Block (Bioneer, Daejeon, Korea). The specificity of the PCR was confirmed by examining the dissociation reaction plot subsequent to real-time RT-PCR. GAPDH served as the constitutive control. Forward and reverse primers were 5^′^-GAATCATTCACCAGGCAAATTG-3^′^ and 5^′^-TCTGTACTGCGGGTGGAACA-3^′^ for COX-2, respectively; 5^′^-GCACCGTCAAGGCTGAGAAC-3^′^ and 5^′^-ATGGTGGTGAAGACGCCAGT−3^′^ for GAPDH, respectively. PCR reactions of each sample were done in triplicate. We took the average value of the control group as internal control. Gene expression levels were calculated and determined using the threshold cycle (C_T_) method (2^-ΔΔCT^ method) [6].

### Statistical analysis

Statistical analysis was performed using the SPSS11.0 software. Differences of the methylation frequencies in the groups were analyzed using the *χ*^2^ test. Differences of mRNA level between the two groups were analyzed using the Student’s *t*-test. Values were expressed as mean + SEM. A *P* value <0.05 was considered statistically significant.

## Results

### Methylation status of cox-2 in endometrial tissues

**Figure 1:**
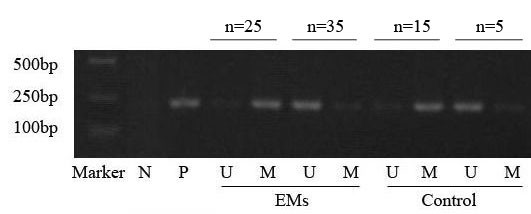
**Representative samples of MSP analyses of DNA samples from the endometrial tissues of the endometriosis and control groups.** Lanes: Marker, molecular weight marker; N, universal unmethylated DNA; P, universal methylated DNA. U/M, PCR products with primers specific for unmethylated and methylated sequences, respectively.

To determine the methylation status of the NF-IL6 site within COX-2 promoter in endometriosis, endometrial tissues from 60 patients with endometriosis and 20 controls without endometriosis were examined by methylation specific PCR (MSP). MSP showed that 41.7% (25/60) of endometrial tissues in the endometriosis group and 75% (15/20) of endometrial tissues in the control group showed the hypermethylation, respectively (*χ*2 = 6.67, *P* = 0.01). Representative examples are shown in Figure 1.

### COX-2 mRNA expression in endometrial tissues

To determine the correlation between DNA methylation and COX-2 expression in endometrial tissues, we evaluated the mRNA level of COX-2 in endometrial tissues between the endometriosis group and the control group by using quantitative real-time RT-PCR. As shown in Table 2, COX-2 mRNA level in endometrial tissues of the endometriosis group was 2.61-fold higher than that in the control group (*P* < 0.01). COX-2 mRNA level of the unmethylated endometrial tissues was 2.39-fold higher than the methylated endometrial tissues in the endometriosis group (*P* < 0.01). Compared to the methylated endometrial tissues, COX-2 mRNA level of the unmethylated endometrial tissues was 2.66-fold higher in the control group (*P* < 0.01). These data suggest that demethylation of COX-2 promoter may be responsible for its abnormally high expression in eutopic endometrium of endometriosis.

**Table 2:** The relationship between the methylation status of COX-2 promoter and its mRNA expression in endometrial tissues (x ± s, n)

	**mRNA level**	**mRNA level(M)**	**mRNA level(U)**
EMs group	2.95 ± 1.35^a^	1.63 ± 0.42^b^	3.90 ± 0.93^b^
	(n = 60)	(n = 25)	(n = 35)
Control group	1.13 ± 0.65^a^	0.80 ± 0.19^c^	2.13 ± 0.49^c^
	(n = 20)	(n = 15)	(n = 5)

^a^*P* < 0.01.

^b^*P* < 0.01.

^c^*P* < 0.01.

mRNA level, mRNA level of COX-2 in endometrial tissues; mRNA level(M), mRNA level of COX-2 in methylated endometrial tissues; mRNA level(U), mRNA level of COX-2 in methylated endometrial tissues.

## Discussion

In the present study, we provided experimental evidence that the frequency of hypermethylation of NF-IL6 site within COX-2 promoter in the endometrium of the endometriosis group was lower than that of the control group (*P* = 0.01). However, COX-2 mRNA level in the endometrium of the endometriosis group was higher than that in the control group (*P* < 0.01). In addition, we found that COX-2 mRNA level of the unmethylated endometrial tissues in endometriosis group was 2.39-fold higher than the methylated endometrial tissues in the same group (*P* < 0.01). Compared to the methylated endometrial tissues in the control group, COX-2 mRNA level of the unmethylated endometrial tissues in the same group was 2.66-fold higher *(P* < 0.01). Our data showed that the hypomethylation of NF-IL6 site within COX-2 promoter may be responsible for the elevated gene expression in eutopic endometrium of endometriosis. Meanwhile, we observed that there was also a significant difference in COX-2 expression levels between unmethylated endometrium of patients with endometriosis and unmethylated endometrium of controls. Thus, the authors speculated that other mechanisms, except hypomethylation COX-2 promoter, may be also participate in elevated COX-2 expression in endometriosis [18].

Endometriosis is an estrogen-dependent gynecological disease causing pelvic pain and infertility. Up to now, the pathophysiology of endometriosis remains an enigma. In the previous study, other workers [9,10] and us [7] have demonstrated that COX-2 was one of the up-regulated genes in the eutopic endometrium of endometriosis. The elevated expression of COX-2 resulted in the production of PGE2, not only regulating cell proliferation, apoptosis, migration, invasion, angiogenesis, and immunomodulation, but also increasing the expression of aromatase and its activity, which lead to higher estradiol formation [19,20]. Additionally, PGE2 is also a potent mediator of pain and inflammation in endometriosis [21]. Thus, COX-2 overexpression that lead to higher levels of prostaglandins may be probably the key mechanism causing endometriosis-related severe dysmenorrheal and inflammation. It was reported that inhibition of COX-2 expression prevented establishment of endometriosis and decreased the size and number of endometriotic lesions in different animal models [22,23]. More importantly, The Food and Drug Administration approved that selective COX-2 inhibitors with milder side effects (for example, rofecoxib and valdecoxib) can be used for the treatment of primary dysmenorrhea, and their long-term administration could potentially reduce the chronic pelvic pain associated with endometriosis [2]. In the present study, we further demonstrated that COX-2 mRNA level in eutopic endometrium of the endometriosis group was significantly higher than that in the control group. Increased COX-2 expression and PGE_2_ production were believed to be strongly correlated with pathophysiology and pathogenesis of endometriosis. However, how COX-2 expression is regulated in detail remains unknown in eutopic endometrium of endometriosis.

Besides the well-known genetic alterations, changes in DNA methylation have been recognized as one of the most common molecular alterations in human neoplasia [24]. Recent studies have indicated that an epigenetic disorder may play a role in the pathophysiology of endometriosis [25]. For instance, Xue *et al.* have reported that DNA hypomethylation of estrogen receptor 2 (ESR2) and steroidogenic factor-1 (SF-1) were responsible for their strikingly elevated level in endometriosis [26,27]. Additionally, DNA hypermethylation and suppressed expression of HOXA10 have been shown in the endometrium of women with endometriosis [28]. However, little is known about the relationship between the methylation status of the CpG island with the COX-2 promoter and COX-2 expression in eutopic endometrium of endometriosis.

There are 51 CpG sites in the promoter region of COX-2 gene from −590 to +186 nt. It is generally accepted that COX-2 expression is regulated predominantly by the activation of three transcription factors: the nuclear factor-kB (NF-kB), the nuclear factor for interleukin-6 expression (NF-IL6), and the cyclic AMP response element (CRE) [29,30]. Song *et al.*[31] reported that the constitutively active COX-2 promoter activity, which was induced by using the NF-IL6 and CRE elements, was completely blocked by heavy methylation of the COX-2 CpG island in gastric carcinoma cells. However, treatment with demethylating agents effectively reactivated the expression of COX-2. Hence, there is a substantial possibility that the aberrant methylation of CpG sites at critical sites may be one of the mechanisms regulating the COX-2 transcription. In particular, Tamura *et al.*[32,33] reported that the NF-IL6 site was involved in inducing COX-2 gene transcription of endometrial stromal cells. Therefore, we examined the methylation status of NF-IL6 site within the COX-2 promoter by methylation specific PCR. Our data showed the frequency of hypermethylation of the NF-IL6 site within COX-2 promoter in eutopic endometrium of the endometriosis group was lower compared to the control group, which was inversely related to the COX-2 expression. Meanwhile, we found that COX-2 mRNA level of the unmethylated endometrial tissues in the endometriosis group and control group was significantly higher than the methylated endometrial tissues of each corresponding group. Although the early, middle, and late secretory phases have very distinct characteristics and COX-2 expression may vary according to the menstrual cycle. However, our data showed that there was no difference between the two groups with respect to the cycle phase. These results showed that the hypomethylation within the promoter of COX-2 may be responsible for the elevated gene expression in eutopic endometrium of endometriosis, which further suggested a possibility that endometriosis was an epigenetic disease.

Several limitations of this study need to be pointed out. First, our study was confined to patients with stages III and IV endometriosis. Whether or not the same methylation alterations also occur in early stages of endometriosis await for further studies. Second, we did not detect the methylation of COX-2 in matched endometriotic tissues, which may further demonstrated the role of aberrant methylation of COX-2 in the pathogenesis of endometriosis.

In conclusions, our present study demonstrated for the first time that DNA hypomethylation of the NF-IL6 site within the promoter of the COX-2 gene may be a key mechanism for its abnormally elevated expression in eutopic endometrium of endometriosis. However, how demethylation of the NF-IL6 site within the COX-2 promoter causes the increased expression of COX-2 in eutopic endometrium of endometriosis needs further study. Further study of similar epigenetic changes may prove to be extremely useful in the diagnosis and treatment of endometriosis in the future.

## Conclusions

To the best of our knowledge, our results indicated for the first time that the hypomethylation of NF-IL6 site within COX-2 promoter may be responsible for the elevated gene expression in eutopic endometrium of endometriosis. Although the biological functions associated with the presence of demethylation-mediated transcriptional activation of the COX-2 gene are not clear, our results showed the DNA hypomethylation of the COX-2 CpG island was involved in the pathogenesis of endometriosis, which also provide an additional evidence that endometriosis ultimately may be an epigenetic disease. Further study of similar epigenetic changes may prove to be extremely useful in the diagnosis and treatment of endometriosis in the future.

## Competing interests

The authors declare that no competing interests exit in the submission of this manuscript, and manuscript is approved by all authors for publication.

## Authors’ contributions

DBW, QC, CYZ, FR, and TL carried out the molecular genetic studies, participated in drafting the manuscript. QC and CYZ performed the statistical analysis. DBW conceived of the study, and participated in its design and coordination and helped to draft the manuscript. All authors read and approved the final manuscript.
